# Supplementary figures and images for: Positive attitudes towards COVID-19 vaccines: A cross-country analysis
Source: PLoS One. 2022 Mar 10;17(3):e0264994. doi: 10.1371/journal.pone.0264994 (PMC8912241; doi:10.1371/journal.pone.0264994)

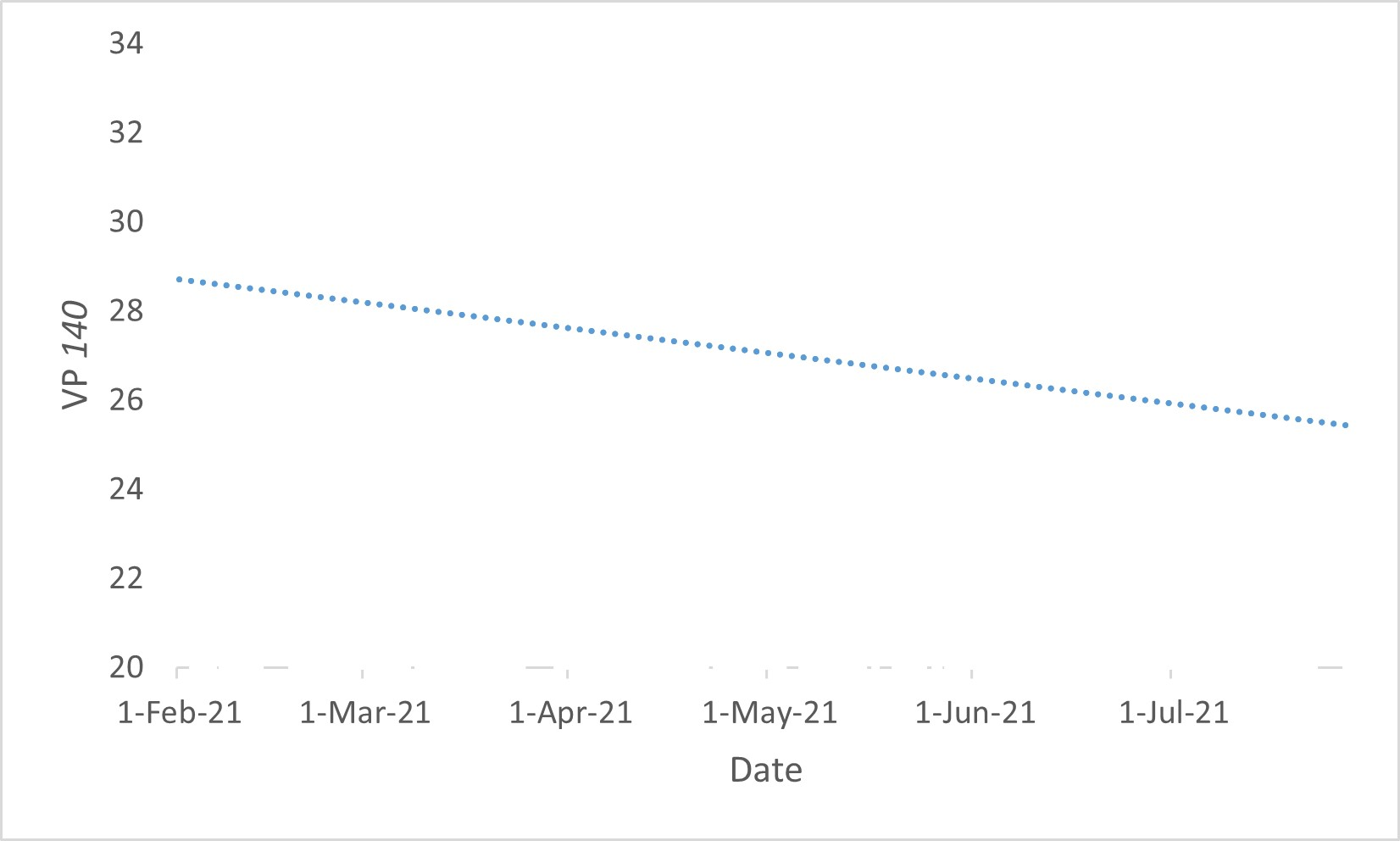

Supplement: S1 Fig — Source: Authors’ calculations. (TIF) [file pone.0264994.s001.tif]

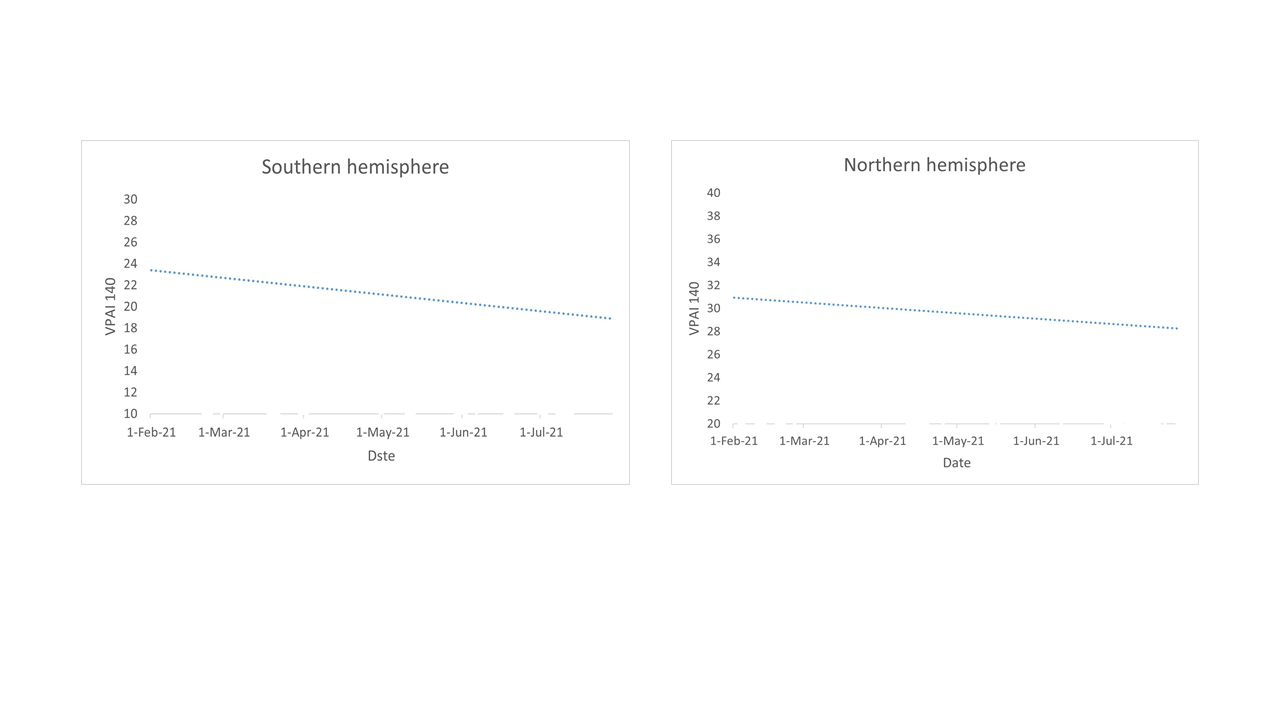

Supplement: S2 Fig — Source: Authors’ calculations. (TIF) [file pone.0264994.s002.tif]

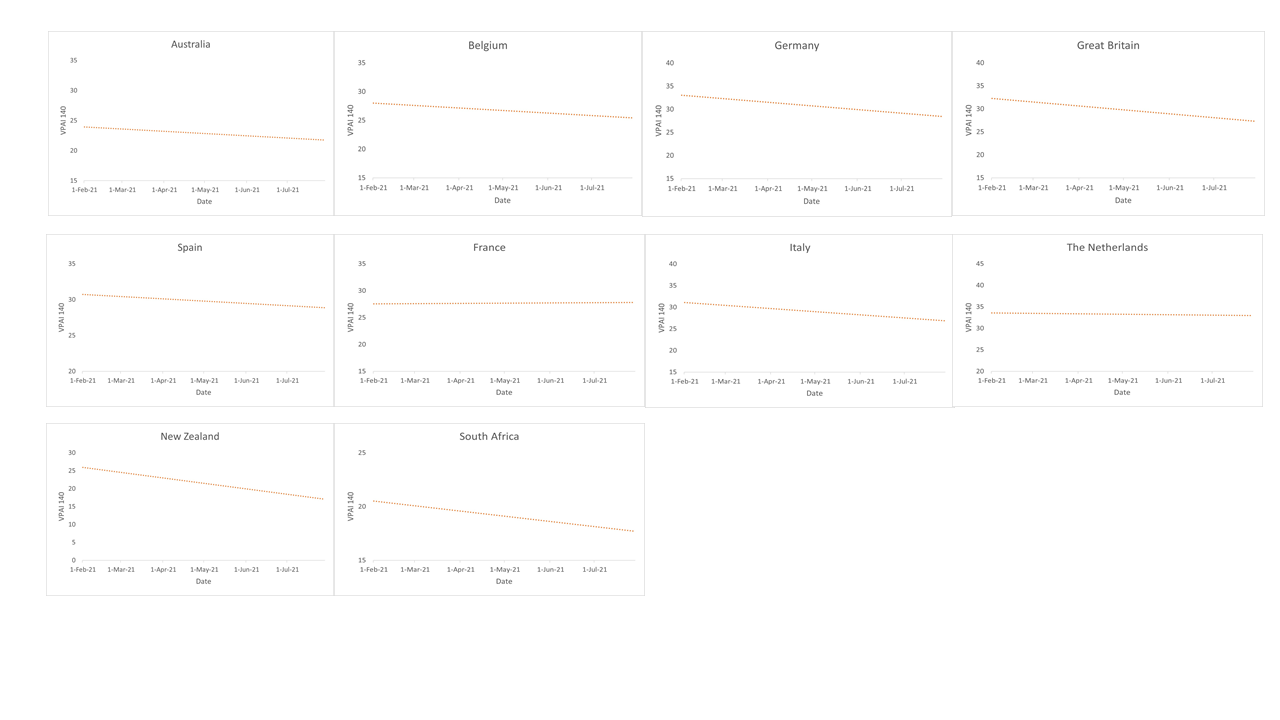

Supplement: S3 Fig — Source: Authors’ calculations. (TIF) [file pone.0264994.s003.tif]

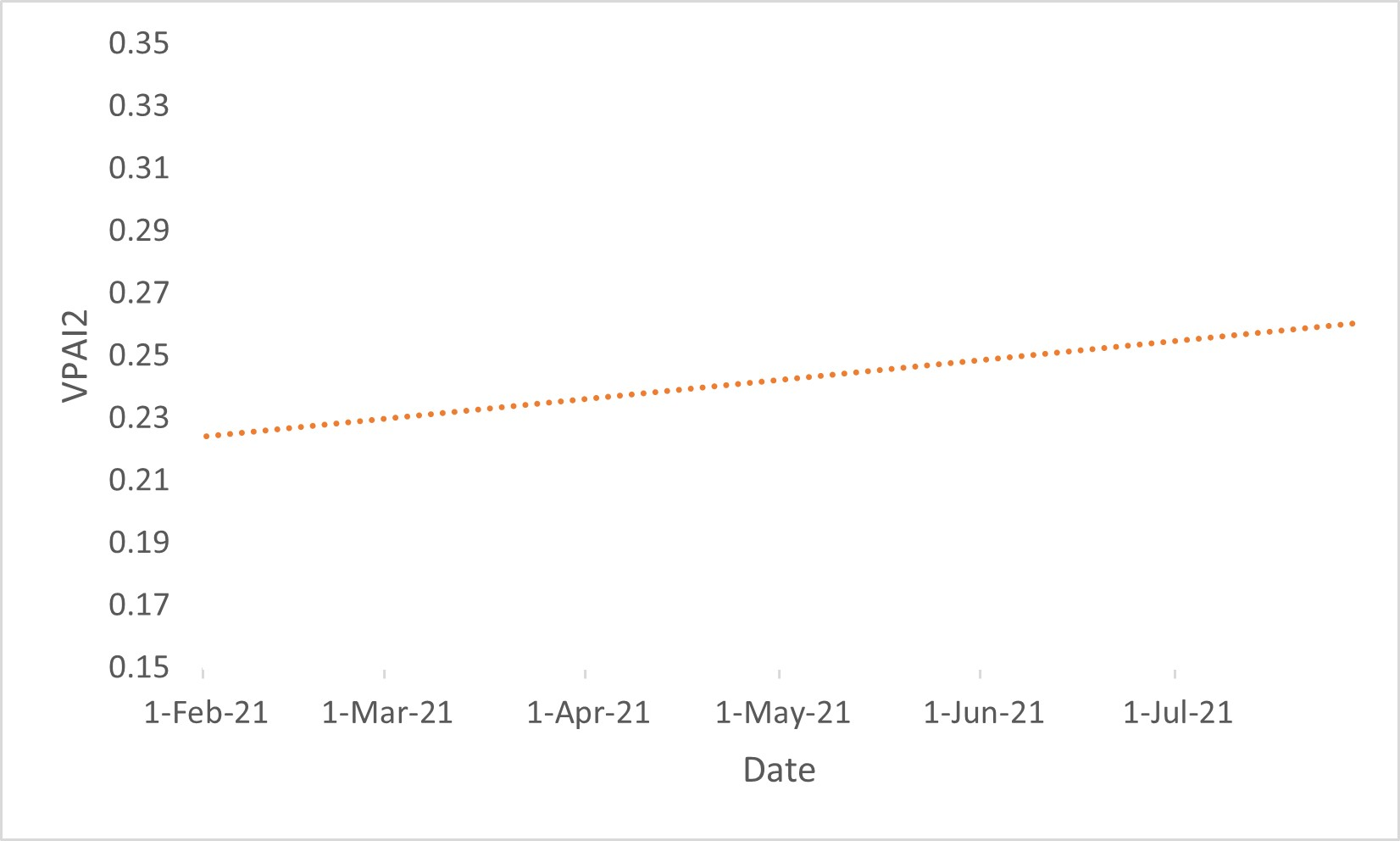

Supplement: S4 Fig — Source: Authors’ calculations. (TIF) [file pone.0264994.s004.tif]

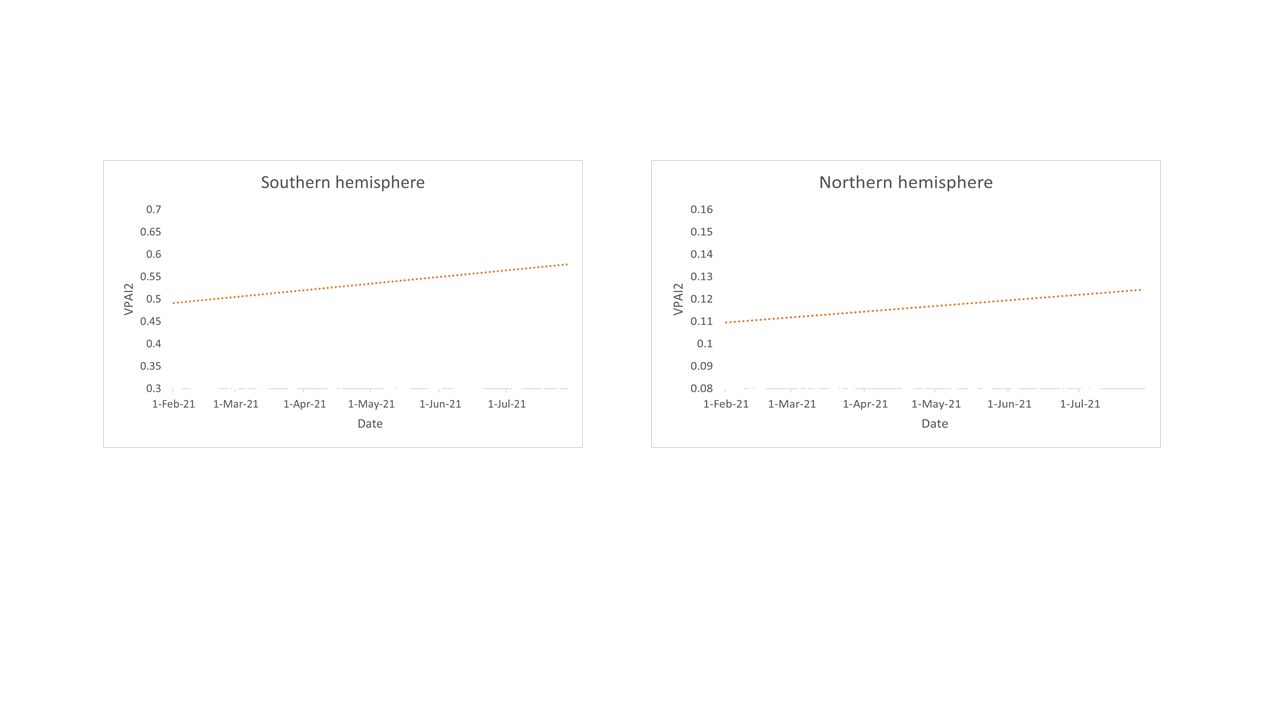

Supplement: S5 Fig — Source: Authors’ calculations. (TIF) [file pone.0264994.s005.tif]

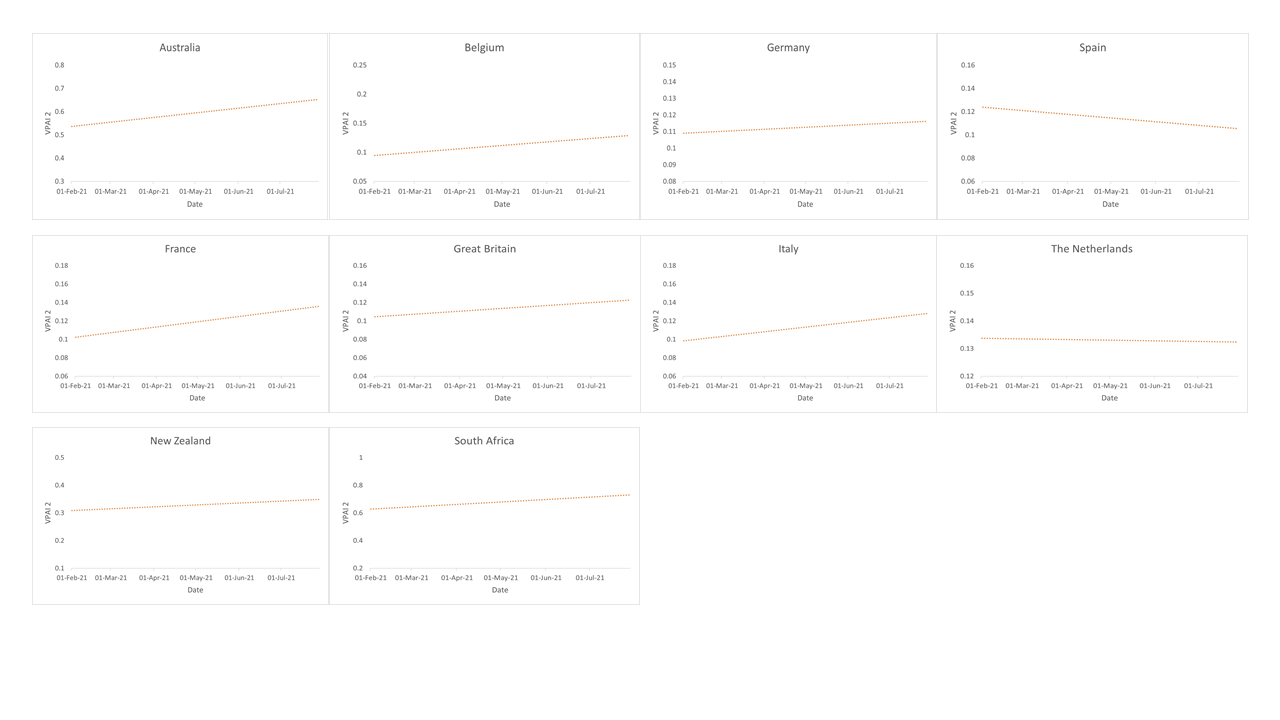

Supplement: S6 Fig — Source: Authors’ calculations. (TIF) [file pone.0264994.s006.tif]
